# Reproducibility of Neurochemical Profile Quantification in Pregenual Cingulate, Anterior Midcingulate, and Bilateral Posterior Insular Subdivisions Measured at 3 Tesla

**DOI:** 10.3389/fnhum.2016.00300

**Published:** 2016-06-21

**Authors:** Nuno M. P. de Matos, Lukas Meier, Michael Wyss, Dieter Meier, Andreas Gutzeit, Dominik A. Ettlin, Mike Brügger

**Affiliations:** ^1^Center of Dental Medicine, University of ZurichZurich, Switzerland; ^2^Institute for Complementary and Integrative Medicine, University Hospital Zurich and University of ZurichZurich, Switzerland; ^3^Seminar for Statistics, ETH ZurichZurich, Switzerland; ^4^Institute for Biomedical Engineering, University of Zurich and ETH ZurichZurich, Switzerland; ^5^Institute of Radiology and Nuclear Medicine, Hirslanden Hospital St. AnnaLucerne, Switzerland

**Keywords:** proton magnetic resonance spectroscopy, anterior midcingulate, pregenual cingulate, posterior Insula, reproducibility, region-specific neurochemistry, functional homogeneity, structural homogeneity

## Abstract

The current report assessed measurement reproducibility of proton magnetic resonance spectroscopy at 3 Tesla in the left and right posterior insular, pregenual anterior cingulate, and anterior midcingulate cortices. Ten healthy male volunteers aged 21–30 years were tested at four different days, of which nine were included in the data analysis. Intra- and inter-subject variability of myo-inositol, creatine, glutamate, total-choline, total-*N*-acetylaspartate, and combined glutamine–glutamate were calculated considering the influence of movement parameters, age, daytime of measurements, and tissue composition. Overall mean intra-/inter-subject variability for all neurochemicals combined revealed small mean coefficients of variation across the four regions: 5.3/9.05% in anterior midcingulate, 6.6/8.84% in pregenual anterior cingulate, 7.3/10.00% in left posterior and 8.2/10.55% in right posterior insula. Head movement, tissue composition and day time revealed no significant explanatory variance contribution suggesting a negligible influence on the data. A strong correlation between Cramer–Rao Lower Bounds (a measure of fitting errors) and the mean intra-subject coefficients of variation (*r* = 0.799, *p* < 0.001) outlined the importance of low fitting errors in order to obtain robust and finally meaningful measurements. The present findings confirm proton magnetic resonance spectroscopy as a reliable tool to measure brain neurochemistry in small subregions of the human brain.

## Introduction

Neuroimaging taught us a lot about functional principles of the human brain with functional magnetic resonance imaging (fMRI) as leading edge technology. Proton magnetic resonance spectroscopy (^1^H-MRS) represents another non-invasive magnetic resonance based method. In contrast to fMRI, which relies on relative changes of blood oxygenation, ^1^H-MRS is able to directly detect and quantify neurochemical components of the living brain. This method provides information about energy metabolism (creatine/phosphocreatine, glucose, and lactate), membrane metabolism and integrity (choline) as well as neurotransmission (glutamate, *N*-acetyl-aspartyl-glutamate, glutamine, GABA, aspartate and glycine). *N*-acetylaspartate and myo-inositol are thought to reflect neuronal and astroglial markers whereas ascorbic acid and glutathione provide the *in vivo* antioxidant profile (Stagg and Rothman, 2013). Compared to fMRI as the mainstay of neuroimaging, ^1^H-MRS thus holds the potential to provide important and complementary (to fMRI) insights with respect to understanding neurochemical related functional principles of the human brain in both, health and disease.

However, application of ^1^H-MRS poses several challenges. Generally, the signals are very minute making spectrum quality and succeeding interpretation of acquired data a critical issue (Kreis, 2004). To compensate for this major constraint, frequently applied volumes of interest – termed voxel – are about 8 ml in size. But, such voxel sizes are often too big to optimally capture relevant brain structures considering their functional and anatomical specifications (Stagg and Rothman, 2013). Exemplary are cingulate and insular brain areas with their corresponding subdivisions. They are crucially involved in a multitude of brain functions reflected in high citation rates across the literature by covering an impressive discipline variety. For example, significant functional contributions of these subareas have been demonstrated in chronic (Denk et al., 2014) and acute pain (Apkarian et al., 2005; Vogt, 2009; Wager et al., 2013), attention deficit hyperactivity disorder (Bush, 2011), anxiety (Damsa et al., 2009), depression (Fountoulakis et al., 2008; Su et al., 2014), and schizophrenia (Takahashi et al., 2003; Haznedar et al., 2004; Costain et al., 2010).

Importantly, a considerable body of literature emphasizes anatomical and functional heterogeneity within those gross cingulate and insular areas. For example, current consensus subdivides the coarse cingulate cortex into six subregions based on cytoarchitectonic and associated functional characteristics (Vogt, 2005, 2009; Dou et al., 2013). In a similar vein, subdividing of the insular cortex into an (at least) anterior and posterior compartment is strongly recommended based on structurally and functionally known attributes (Varnavas and Grand, 1999; Kurth et al., 2010a,b; Stephani et al., 2011; Mazzola et al., 2012a,b; Nieuwenhuys, 2012).

For obtaining more meaningful ^1^H-MRS measures, voxels should therefore cover functional homogeneous cingulate and insular subregions as distinctly as possible (Dou et al., 2013; Gutzeit et al., 2013).

The critical points are that such subregion dimensions clearly are below 8 ml, and applicable ^1^H-MRS volumes are limited to cuboid shapes. Those two aspects hamper adequate anatomical coverage of the target areas because gyrification and structure contours are mostly bent. Consequently, voxels should be even smaller than the actual target areas to ensure appropriate anatomical (and functional) specificity.

Additionally, besides covering the desired gray matter (GM), they also include white matter (WM) and cerebrospinal fluid (CSF). Hence, measured signals derived from all these tissue components induce another source of uncertainty regarding meaningful interpretation of the underlying area’s specific neurochemistry (Stagg and Rothman, 2013; in fMRI studies, this issue is circumvented by specific tissue segmentation strategies implemented in all analysis software packages, but this practice is not yet common in ^1^H-MRS reports). Therefore, when applying such small voxels in an attempt to optimally capture relevant brain subregions, it must be clarified as to whether quality aspects of ^1^H-MRS measurements are affected in order to gain the appropriate information with respect to the neurochemical milieu within the target areas.

This study was conceptualized to explore ^1^H-MRS measurement reproducibility in anatomically and functionally homogeneous subregions of cingulate and insular cortices, all substantially below 8 ml. Areas investigated were: pregenual anterior cingulate cortex (pgACC), anterior midcingulate cortex (aMCC), as well as left and right posterior insular cortex (pIL and pIR). Measurements were conducted using standard MR-equipment and ^1^H-MRS sequences at 3 Tesla in order to allow application and replication in commonly available clinical and research settings.

## Materials and Methods

The present study was approved by the local ethics committee and conducted according to the Declaration of Helsinki.

### Subjects and Experimental Procedure

Ten healthy male volunteers (mean age = 25.3, range: 21–30) were recruited for the study. Exclusion criteria were general contraindications for MR, neurologic and psychiatric diseases as well as any form of pain conditions. Every subject attended four scan visits on different days (mean intersession interval = 11.03 days; *SD* = 10.98; range = 1–42 days). All acquisitions (except for one measurement which was performed at 2:00 PM) were conducted at one of four time slots in the afternoon and evening (Time slot 1: 4:00–5:30 PM; Time slot 2: 5:30–7:00 PM; Time slot 3: 7:00–8:30 PM; Time slot 4: 8:30–10:00 PM). The overall mean starting time was 5:47 PM (*SD* = 1 h 44 min).

Subjects received detailed information about the experimental procedure, aim of the study and provided written informed consent. Participants were informed in detail about the importance of remaining as motionless as possible during the scan sessions. They were further instructed not to consume alcohol, analgesic medication and other drugs on examination days as well as to be fed.

### Scanning

Subjects were comfortably positioned in the scanner in a supine position. The Pearltec Crania (Pearltec AG, Schlieren/Zurich, Switzerland) head fixation system was applied for an optimal and comfortable head fixation. MR measurements consisted of a survey scan, a T1-weighted anatomical scan for precise voxel placement and tissue segmentation (duration = 7 min 29 s) followed by four consecutive single-voxel MRS measurements in the four target areas (duration = approximately 6 min each). To consider possible head movements, an adapted short T2-weighted image (duration = 21 s) was recorded before and after each MRS sequence in order to estimate the extent of voxel displacements. The measurement order of the brain regions was alternated between the four measurement days to account for measurement order effects and frequency drifts due to heating and other time dependent MR-system changes. The total duration of the MR measurements was approximately 45 min.

### MR Protocols

MR experiments were performed on a 3-Tesla MR unit (Ingenia, Release 4.1.3, Philips Healthcare, Best, The Netherlands) utilizing a dS-SENSE 15-channel head coil (Philips Healthcare, Best, The Netherlands). Parameter of the T1-weighted turbo gradient echo sequence for voxel placement and segmentation were: TR/TE = 8.1/3.7 ms; flip angle = 8°; FOV_FHxAP_ = 240 mm × 240 mm; 160 slices; recorded voxel size = 1 mm isometric. To calculate possible head movements, a short T2-weighted turbo spin-echo sequence (<30 s) was performed in axial orientation with the following parameters: TR/TE = 3000/80 ms, flip angle = 90°; FOV_APxRL_ = 230 mm × 182 mm; resulting voxel size = 1 mm × 1 mm × 4 mm.

For the MR spectra acquisition, a conventional single-voxel PRESS (point-resolved spectroscopy) sequence (TR/TE = 2500/32 ms; data points = 1024; sample frequency = 2000 Hz; readout duration = 512 ms; number of acquisitions = 128) was used. A water-unsuppressed spectrum recorded with 16 acquisitions was acquired prior to the main spectrum for *post hoc* spectral corrections. Water suppression was achieved using a VAPOR (variable pulse power and optimized relaxation delays) scheme. An implemented automatic second-order projection-based shimming routine (Gruetter, 1993) was used for the reduction of *B*_0_-inhomogeneities.

**FIGURE 1:**
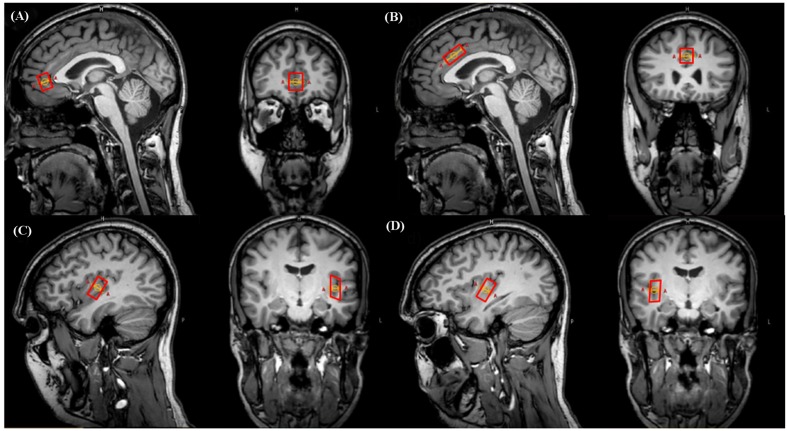
**Representative illustration of voxel positionings on sagittal and coronal plane images in a single subject.** **(A)** Pregenual anterior cingulate cortex (pgACC), **(B)** anterior midcingulate cortex (aMCC), **(C)** left posterior insula (pIL), **(D)** right posterior insula (pIR).

The pgACC and aMCC voxels were carefully placed according to suggestions from specific literature (Vogt, 2005, 2009). The respective dimensions were: pgACC 12 mm × 16 mm × 18 mm (AP × LR × FH; 3.46 ml), aMCC 23 mm × 15 mm × 12 mm (4.14 ml). Voxel placements for left and right posterior insular subdivisions were based on neuro-anatomic and functional work (Varnavas and Grand, 1999; Kurth et al., 2010a; Mazzola et al., 2012a; Nieuwenhuys, 2012). They were placed posterior and aligned to the insular central sulcus including the gyri longi leading to voxel dimensions of 12 mm × 11 mm × 23 mm (3.04 ml). Voxel placement was always performed by the same person (NM) (**Figure 1**).

### Quantification of Spectroscopic Data

Spectroscopic data were quantified with LCModel (Provencher, 1993) using a basis set for an echo time of 32 ms including the following *in vitro* neurochemical model spectra: *N*-acetylaspartate (NAA), *N*-acetyl-aspartyl-glutamate (NAAG), glutamate (Glu), glutamine (Gln), γ-aminobutyric acid (GABA), glutathione (GSH), glycerophosphocholine (GPC), phosphocholine (PCh), creatine (Cre), myo-Inositol (mI), ethanolamine and phosphorylethanolamine (PE), aspartate (Asp), glucose (Glc), lactate (Lac), scyllo-inositol (sI), and taurine (Tau). Substances with strongly overlapping spectra were combined to single spectra in order to improve quantification accuracy: Glu + Gln = Glx, GPC + PCh = tCho and NAA + NAAG = tNAA. The chemical shift range for the fitting procedure was set to 4.0–0.2 ppm. Fitted neurochemical concentrations were scaled to the internal water signal obtained from the corresponding water-unsuppressed spectrum acquisition. For all subsequent analysis steps we included the following neurochemicals, which demonstrated Cramér–Rao lower bounds (CRLB) < 20%: mI, Cre, Glu, tCho, tNAA, Glx. A correction was applied to account for the fact that metabolic signals originate mainly from WM and GM but not from CSF (McLean et al., 2000). For this purpose, the T1-weighted anatomical images were segmented into brain tissue compartments (GM, WM, and CSF) using the segmentation routine included in the SPM8 software package^1^ running on MATLAB 2013b (MathWorks, Natick, MA, USA). The calculation of tissue fractions in the applied spectroscopic voxels were performed using a self-written MATLAB script. The neurochemical concentrations (*C*_0_) were corrected applying the following equation using the estimated fraction of CSF (*F*_CSF_; McLean et al., 2000):

$$C=C_{0}*\left(1/\left(1-F_{CSF}\right)\right)$$

### Head Movement Analysis

Anatomical T2-weighted scans were analyzed to track possible head movements between spectroscopic acquisitions using the realignment function included in the SPM8 software package^1^ running on MATLAB 2013b (MathWorks, Natick, MA, USA). Defining the first anatomical scan as reference, following scans were realigned to it providing estimates for translational (mm) and rotational (deg) displacements in the *x*-, *y*-, and *z*-axis over time.

In the next step, maximum displacements in the three translational and rotational orientations were determined for each individual session and then averaged over all sessions. This approach provides a more stringent evaluation of possible movement interference compared to mean intra-session displacements, as it represents more accurate strong head movements.

### Statistical and Reproducibility Analyses

Statistical analyses were carried out with the SPSS Statistics 22 software package (IBM Corp., Armonk, NY, USA).

Mean neurochemical concentrations between the four spectroscopy acquisitions were compared with a repeated measure ANOVA (rmA).

In addition, reproducibility was determined by estimating the magnitude of intra- and inter-subject variability by means of coefficients of variation (CV, calculated as *SD*/Mean^∗^100).

Coefficients of variation is preferable to other dispersion measures due to its independence of the underlying scale (Lovie, 2005).

Measures of reliability like the intra-class correlation (ICC) have also been used in MRS studies. The problem with this measure is its dependence on variation of a trait (like true differences in neurochemical concentrations between persons) in the measured populations, thus impeding a direct comparison of ICC values from different studies (Müller and Büttner, 1994; Henriques et al., 2013). In this vein, a heterogeneous population could yield higher (more reliable) ICC than a homogeneous population despite similar intra-subject variability. We therefore decided not to apply this measure in our study.

Inter-subject variability was calculated by randomly selecting one time point from the four repeated measures of all participants and calculating mean and standard deviation. This step was repeated 5000 times to account for different combination possibilities. Afterward, the 5000 means and SDs were averaged. The resulting means and SDs were used to calculate the inter-subject CV.

Variations in GM/WM-ratio, daytime of participation and magnitude of between session movements contribute to intra-subject variability (Kreis, 2004; Soreni et al., 2006). Despite the applied precaution to control such effects, they might still have influenced variability. In order to estimate their effects, an ANCOVA with participants and regions as fixed effects (to account for variance attributable to participant and region characteristics) was calculated with GM/WM-ratio, daytime of participation and the absolute voxel displacement (*d*) as covariates. The vector representing the voxel displacement was calculated as follows:

$$d=\sqrt{X^{2}+Y^{2}+Z^{2}}$$

*X*, *Y*, and *Z* correspond to the three directions of the voxel displacement; therefore *d* is the vector of the three displacement directions describing the absolute voxel displacement.

## Results

One participant had to be excluded due to problems with the tissue segmentation procedure, resulting in nine full data sets with a total of 36 spectra per region.

### Movement

Results from the head movement analysis are summarized in **Table 1**. The displacement during the sessions was minute, with maximal shifts of 1.5 mm in the *X*-axis, 1.51 mm in the *Y*-axis, and 1.43 mm in the *Z*-axis [mean of maximum displacements: 0.31 ± 0.23 mm (*X*); 0.43 ± 0.24 mm (*Y*); 0.77 ± 0.37 mm (*Z*)]. The estimated maximal rotational changes were negligible with maximum rotations of 2.51° in the pitch, 0.79° in the roll, and 1.78° in the yaw-angle. We therefore expect head movement to be an insignificant source of variation.

**Table 1:** Mean, standard deviation (SD), and range of translational (mm) and rotational (deg) movement estimation.

	Translation (mm)			Rotation (Degrees)		
		
	*X*-axis	*Y*-axis	*Z*-axis	Pitch	Roll	Yaw
Mean ± SD	0.31 ± 0.23	0.43 ± 0.24	0.77 ± 0.37	0.44 ± 0.48°	0.30 ± 0.19°	0.55 ± 0.40°
Range	0.07–1.28	0.08–1.51	0.28–1.43	0.08–2.51°	0.04–0.79°	0.1–1.78°


### Spectral Quality, Fitting Errors, and Tissue Fractions

One important aspect of chosen voxel dimensions was the maximization of gray matter tissue portion to enhance tissue specificity and account for partial volume effects. The results from the tissue segmentation procedure are shown in **Table 2**. Highest GM contents were found in the pgACC voxels (80.85%), the smallest in the aMCC voxels (74.58%). Accordingly, WM percentages were small with mean values between 8.48% (aMCC) and 4.29% (pgACC). The remaining contributions from CSF were between 16.86% (aMCC) and 14.04% (pIL) but are insignificant due to the applied CSF-correction.

**Table 2:** Spectral quality and tissue fractions in the voxels (mean ± SD).

	aMCC	pgACC	pIL	pIR
Spectral quality:
SNR	16 ± 1.79	12.78 ± 1.71	11.94 ± 1.37	11.28 ± 1.43
LW_H20_ (Hz)	0.0532 ± 0.0004	0.0711 ± 0.010	0.0513 ± 0.0025	0.0514 ± 0.0020
Voxel size:	4.14 cc	3.46 cc	3.04 cc	3.04 cc
Tissue composition:
GM	74.58 ± 2.36%	80.85 ± 2.07%	77.62 ± 2.89%	77.68 ± 4.38%
WM	8.48 ± 3.10%	4.29 ± 1.89%	8.24 ± 2.01%	7.53 ± 1.99%
CSF	16.86 ± 3.58%	14.77 ± 2.98%	14.04 ± 2.75%	14.70 ± 0.65%


*SNR, signal-to-noise ratio; LW_*H20*_, linewidth of the water peak (in Hz); GM, gray matter; WM, white matter; CSF, cerebrospinal fluid; aMCC, anterior midcingulate cortex; pgACC, pregenual anterior cingulate cortex; pIL/pIR, left/right posterior insula. Please note that the sum of GM, WM and CSF can deviate form 100 due to rounding errors and segmentation dependent quantification uncertainties.*

Differences in tissue percentages between the four sessions could increase intra-subject variability. However, intra-subject differences of GM fractions between the four measurement repetitions were minimal with a mean CV of 1.83% over all participants and regions. We were able to record high quality spectra using small voxel sizes (3.04–4.14 ml) and moderate number of acquisitions (128). The level of SNR in the spectra was between 11.28 (pIR) and 16.00 (aMCC). The mean linewidth of the unsuppressed water signal was <0.054 ppm for all regions except for the pgACC (0.071 ppm).

The good data quality resulted in overall small CRLBs although higher fitting errors were observed in the posterior insulae compared to aMCC and pgACC (**Table 3**).

**Table 3:** Characteristics of neurochemical concentrations, fitting errors (CRLB), and variability (CV) per region.

	aMCC						pgACC					
		
	mI	Cre	Glu	tCho	tNAA	Glx	mI	Cre	Glu	tCho	tNAA	Glx
Mean CRLB [%]^a^	6.7	4.2	6.9	4.5	3.7	6.1	6.5	4.2	6.6	4.9	4.4	6.0
(range)	5–11	4–5	6–8	3–6	3–4	5–7	5–8	3–5	5–8	4–6	4–6	5–7
Mean concentrations (AU)^b^	4.4	5.3	10.2	1.8	8.1	14.6	4.9	5.7	11.2	2.0	8.0	15.9
Intra-subject SD^c^	0.36	0.25	0.48	0.11	0.27	0.70	0.41	0.32	0.70	0.17	0.36	0.97
(CV%)	8.8	4.7	4.7	5.8	3.4	4.8	8.2	5.8	6.2	8.7	4.4	6.1
Inter-subject-SD	0.63	0.47	0.70	0.16	0.66	1.10	0.58	0.41	1.06	0.18	0.62	1.23
(CV%)	14.3	8.8	6.9	8.7	8.1	7.5	11.8	7.3	9.5	8.9	7.7	7.8

	**pIL**						**pIR**					
		
	**mI**	**Cre**	**Glu**	**tCho**	**tNAA**	**Glx**	**mI**	**Cre**	**Glu**	**tCho**	**tNAA**	**Glx**

Mean CRLB[%]^a^	8.7	4.6	8.6	5.7	4.6	7.6	9.1	5.0	9.1	6.6	4.6	8.1
(range)	7–20	4–5	7–12	4–7	4–6	6–9	6–16	4–6	7–12	5–10	4–7	6–11
Mean concentrations (AU)^b^	4.4	5.9	10.4	1.8	8.2	14.7	4.6	5.9	10.6	1.7	8.3	15.1
Intra-subject SD^c^	0.39	0.27	1.06	0.12	0.35	1.24	0.44	0.48	1.05	0.12	0.47	1.35
(CV%)^d^	9.9	4.5	10.1	6.6	4.2	8.4	9.6	7.9	10.2	7.0	5.7	8.9
Inter-subject-SD	0.74	0.35	1.16	0.18	0.49	1.51	0.65	0.57	1.25	0.17	0.58	1.65
(CV%)	16.7	5.9	11.1	10.1	6.0	10.2	14.0	9.7	11.8	10.0	6.9	10.9%


*^a^CRLB, Cramer-Rao Lower Bound. CRLB are estimates of the fitting errors provided by LCModel.*

*^b^AU, arbitrary units.*

*^c^Intra-subject SDs were calculated as the average of individual SD from the four sessions.*

*^d^Intra-subject CVs were calculated as the average of individual CV from the four sessions.*

*aMCC, anterior midcingulate cortex; pgACC, pregenual anterior cingulate cortex; pIL/pIR, posterior insula left/right; mI, myo-inositol; Cre, creatine; Glu, glutamate; tCho, total choline; tNAA, total N-acetyl aspartate; Glx, glutamate + glutamine.*

### Reproducibility

**FIGURE 2:**
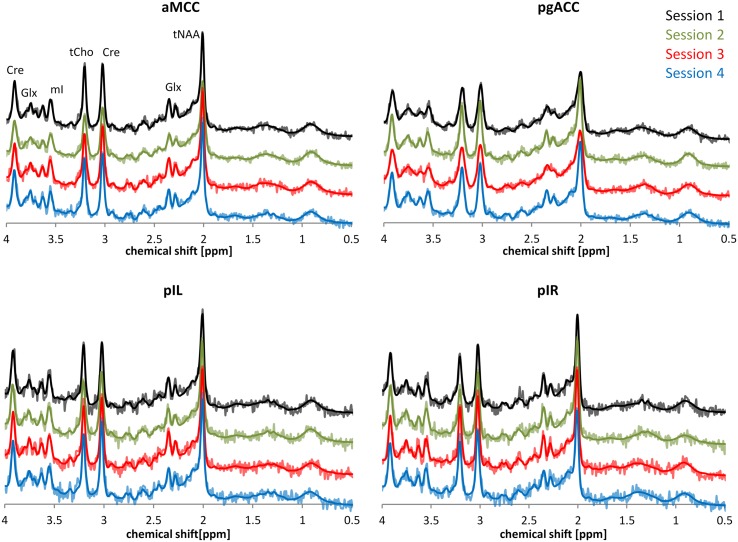
**Exemplary depiction of spectral intra-subject variability based on representative spectra of one participant.** The time interval between each of the four sessions was 7 days. Spectra belonging to the four sessions are illustrated using different colors. Dark lines in the spectra represent the LCModel fit; brighter lines show the underlying raw data.

**FIGURE 3:**
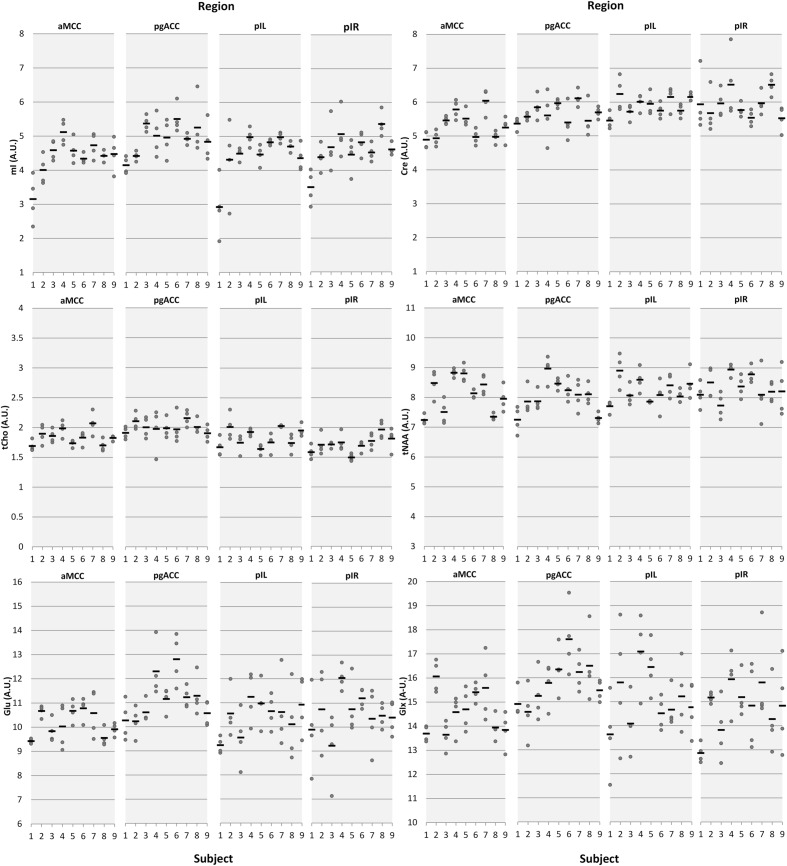
**Intra- and inter-subject variations of neurochemical concentrations illustrated by means of dot plots.** Dots represent individual measurements; the horizontal black bars represent the mean of the four measurement days. The *x*-axis represents the subject number, the *y*-axis represents the metabolite concentrations in arbitrary units (AU) aMCC, anterior midcingulate cortex; pgACC, pregenual anterior cingulate cortex; pIL/pIR, posterior insula left/right.

**Figure 2** provides a descriptive visual illustration of the four repeatedly acquired spectra in a given subject. **Figure 3** illustrates the overview of intra- and inter-subject variations of neurochemical concentrations over all subjects. Differences in neurochemical concentrations between the four intra-individual measurements were analyzed by means of an ANOVA for repeated measures and no significant differences were found.

Estimates of intra-subject and inter-subject variability are summarized in **Table 3**. The overall intra-subject variability was low with mean CV per region over all reported neurochemicals of 5.3% (aMCC), 6.6% (pgACC), 7.3% (pIL), and 8.2% (pIR). Over all regions, the least variation was observed for tNAA (4.42%) whereas the largest variation was observed for mI (9.12%).

Inter-subject variability showed a similar pattern with mean CV over all reported neurochemicals of 9.05% (aMCC), 8.84% (pgACC), 10.00% (pIL), and 10.55% (pIR). tNAA showed the least mean variability (CV 7.18%) over all subareas whereas mI had the largest mean dispersion (CV = 14.23%). Differences between intra- and inter-subject. CV were smaller than expected, with a mean difference of just 2.75%, pointing toward minimal subject-specific differences in neurochemical concentrations. The small inter-subject effect might be attributable to the homogeneous group of participants tested.

To test different sources of intra-subject-variability (GM/WM ratio, daytime of participation and head movements), an ANCOVA with participants and regions as fixed effects was conducted using these variables as covariates. This calculation revealed no significant explanatory contribution of variance by the covariates confirming their negligible effect on the data.

**FIGURE 4:**
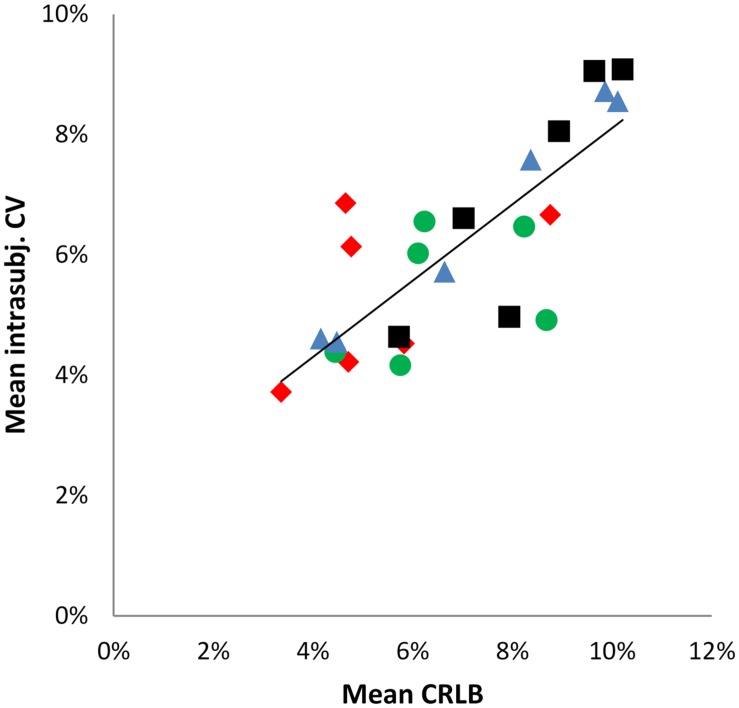
**Scatter plot illustrating the positive correlation between mean intra-subject CV and mean CRLBs.** Data points represent the six neurochemicals, data point shape and colors correspond to the four brain regions. *r* = 0.779; *R*^2^ = 0.6382. 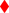 = aMCC, 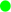 = pgACC, 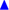 = pIL, 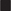 = pIR.

In order to estimate the impact of the fitting error expressed as CRLBs, a Pearson correlation analysis was performed between mean intra-subject CV and mean CRLB over all neurochemicals for the four regions (**Figure 4**) (according to Geurts et al., 2004), revealing a significant positive correlation (*r* = 0.799, *p* < 0.001).

## Discussion

The current report explored reproducibility of ^1^H-MRS at 3 T in the following subregions of the cingulate and insular cortices: pgACC, aMCC cortices as well as left and right posterior insula (pIL and pIR). Results confirm that important neurochemical compounds are reliably quantifiable intra- and inter-subjectively over four measurement sessions on different days. In favor of a better overview, the discussion will be categorized into four sections: (1) Specificity, (2) data quality, (3) intra-subject variation and (4) inter-subject variation. Afterward, the results will be discussed in a broader context.

### Specificity

In the present context, the term specificity encompasses (1) the accurate positioning of the voxels within the anatomical borders of aMCC, pgACC, pIL, and pIR, (2) the achievement of a high GM percentage and (3) is further referred to the exact voxel repositioning across the four measurement days.

Regarding the point (1), to remain as precise as possible within the regions specific anatomical borders, applied voxel sizes resulted in 3.46/4.14 ml for pgACC/aMCC and 3.04 for pIL/pIR. In terms of both cingulate subareas, we followed the classification scheme suggested by Vogt and colleagues (see the “Materials and Methods” section “Definition and Placement of Voxels”) as practically as possible. However, note that according to *ex vivo* anatomical topography, the unilateral pgACC possesses volumes of about 6.4 ml, and the unilateral aMCC volumes of about 4.3 ml (talairach space; Vogt, 2009). In order to remain clearly within the anatomical borders together with the limitation of cuboid voxel shapes, the revealed volumes in this report were smaller with 3.46 for the pgACC and 4.14 for the aMCC subareas. Particularly, the pgACC voxel is much smaller than the reported *ex vivo* anatomy based volumes reasoning in the contour of this subdivision: it is heavily bent and hence we were coerced to reduce voxel dimensions in order to clearly remain within the pgACC borders. With respect to the aMCC, it was possible to cover the effective shape of this subregion rather accurately as it is a less bent cingulate subarea (see **Figure 1**).

For the pIL and pIR, sizes of 3.04 ml were finally used to approximate the anatomical borders as exactly as possible following several anatomical and functional reports (see the “Materials and Methods” section “Definition and Placement of Voxels”). Please note that the remaining voxels of the posterior insulae contained (however, to a small extent) tissue from surrounding areas (see **Figure 1**) due to gyrification and anatomical shape of adjacent gyri and temporal located areas. This situation combined with the cuboid voxel issue impeded an even more precise voxel positioning. Higher anatomical specificity might be attainable by further reducing voxel dimensions, however, with critical SNR costs, requiring very long acquisition times hardly applicable for *in vivo* measurements. Other specificity enhancement strategies are under development and partly usable, i.e., arbitrary voxel shapes. This strategy allows drawing of the region of interest along its exact contours, thus a high spatial selectivity can be theoretically achieved (Weber-Fahr et al., 2009; Snyder et al., 2012).

Regarding the point (2), results from the tissue segmentation analysis confirmed high GM fractions in our volumes with percentages of 74.58% (aMCC), 80.85% (pgACC), 77.62% (pIL), and 77.68% (pIR). Being able to delineate high GM contents are of particular importance for a multitude of basic neuroscientific/clinical questions as it increases sensitivity for the observation of neurochemical processes associated with neural activity (e.g., Synaptic transmission, energy metabolism, etc., Mangia et al., 2007a,b; Gussew et al., 2010) and GM-specific structural changes (e.g., loss of neurons indicated by decreases of NAA; Stagg and Rothman, 2013).

Regarding the point (3) can only be indirectly addressed, but small mean GM variations in the voxels (pgACC: 1.61%, aMCC: 1.44%, pIL: 2.15%, pIR: 2.11%) across the four measurement days over all subjects indicate fairly small differences in voxel repositioning.

### Data Quality

The quality properties of acquired spectra depend on a multitude of variables (for review, see Kreis, 2004). Main criteria are peak linewidth and SNR which are directly linked to voxel sizes. The smaller the voxel, the more negatively SNR is influenced, leading to lower spectral quality. In this report, voxel sizes from 3.04–4 to 14 ml were applied and revealed acceptable to good spectral quality reflected in SNRs from 11.28 (pIR) to 16.00 (aMCC). On the contrary, spectral resolution can benefit from reduced voxel dimensions by a facilitation of *B*_0_-shimming. An enhanced shim allows for narrower and higher peaks enhancing neurochemical peak discrimination and resulting in better SNR (Kreis, 2004). Therefore, SNR loss due to reduced voxel dimensions can be partly compensated by improved shimming (Hock et al., 2013a).

In our study, the revealed spectra resolution (reflected by mean water peak linewidths) was excellent within aMCC (0.0532 ppm), pIL (0.0513 ppm), and pIR (0.0514 ppm) but less within pgACC (0.0711 ppm). pgACC water peak linewidths were more than 25% broader compared to the other three regions. Similar observations have been reported by Dou et al. (2013), using an almost identical pgACC voxel positioning and size. We hypothesize that this concomitant observation might be attributed to interfering effects caused by arteria cerebri anterior (subcallosal artery) pulsation, clearly affecting the accurate shimming of the small pgACC voxel. Furthermore, varying frequency changes in local static magnetic field (*B*_0_) during spectral acquisition, mainly evoked by arterial pulsation, could additionally cause broadening of the peak linewidths (Hock et al., 2013b; Stagg and Rothman, 2013). In this context, techniques applied in spinal cord ^1^H-MRS – such as frequency alignment of single free induction decays before averaging, or cardiac triggering – can improve spectral resolution obtained from pgACC and other critically located brain areas (Hock et al., 2013a,b).

Generally, the good spectral quality resulted in low overall estimated fitting errors for the reported neurochemicals and regions. Mean CRLB were below 10% (thus lower than the typical consensus of 20%), although the fitting errors were larger for insular cortex subregions (**Table 3**). This dissimilarity is probably due to the smaller voxel sizes (3.04 ml) and therefore slightly lower signal intensities compared to the cingulate subregions (3.46/4.14 ml). Important to mention is that higher number of averages per region (although resulting in a longer acquisition time, which is not always feasible) results in increased SNR. This approach could compensate for signal drop accompanied by small voxel sizes thus lowering CRLB values. In this vein, to enhance spectral quality (and therefore reproducibility) it is favorable to combine small voxel dimensions and increased numbers of averages (instead of the usage of big voxels) to get both, narrow peak linewidths and high SNR (Kreis, 2004; Nacewicz et al., 2012).

### Intra-subject Variability

Results showed small intra-subject variation with mean CVs of 5.3% (aMCC), 6.6% (pgACC), 7.3% (pIL), and 8.2% (pIR) over all reported neurochemicals, indicating robust within-subject measurement reproducibility. Intra-subject variability is a conglomerate of various variance sources. It can be influenced by the software quantification error, inaccurate voxel positioning/repositioning, individual biological variation between measurements (as daytime of participation) and head movement (Kreis, 2004; Stagg and Rothman, 2013). Thus considerable effort was put into controlling variance from the aforementioned factors. As a consequence, head movements were minute, variation of daytime of participation was acceptable. An ANCOVA with participants and region as fixed effects and GM/WM ratio, daytime of participation and head movements as covariates showed no significant explanatory contribution (by the covariates), confirming their rather negligible impact on the data. Otherwise, the fitting error (expressed as CRLB) seems to be a crucial source of intra-subject variance in our data demonstrated by a strong positive correlation (*r* = 0.799) between mean intra-subject CV and mean CRLB over all neurochemicals/regions.

An extreme example for the relationship between intra-subject CV and CRLB can be found in the dot-plot illustrating each single neurochemical concentration (**Figure 3**). Focusing on the first two participants, obviously higher intra-subject variation of pIL mI concentrations compared to the other subjects is depicted. Analysis of fitting errors showed the highest CRLB exactly for these two subjects (subject 1 [mean, range]: 13.5%, 9–20%); subject 2: 10.5%, 7–16%; group average of subject 3–9: 8%, 7–10%); corroborating our suggestion that CRLB mainly accounts for measured fluctuations in some neurochemicals/regions. We do not have a concise explanation for the outcome of these two participants as all assessed co-variables were unobtrusive.

### Inter-subject Variability

Inter-subject variations with mean CVs of 9.05% (aMCC), 8.85% (pgACC), 10.00% (pIL), and 10.55% (pIR) were observed. Surprisingly, presented data indicate small differences between inter- and intra-subject CVs with mean CV-difference of 2.75%. We think that the main reason for this observation is the homogeneous sample tested as all participants were male and between 20 and 30 years old. Previous studies showed significant age and gender effects on neurochemical concentrations (Grachev and Apkarian, 2000, 2001; Haga et al., 2009), thus our approach in keeping age and gender within a tight range provides a probable explanation for the small differences in present data. This factor should generally be taken into account more rigorously in experimental settings, as effects can be attributed to age dependent alterations in neurochemical signatures rather than to the experimental variation itself (Gutzeit et al., 2013; Stagg and Rothman, 2013).

## General Discussion of the Results Within the Literature Context

In principle, each ^1^H-MRS reproducibility study is inherently unique due to differences in applied MR scanner equipment and sequences, brain areas measured, number or participants tested, number of measurement sessions performed and the type of statistics finally reported. It is thus advisable to cautiously compare results across publications as no conclusive reproducibility criteria exist (Stagg and Rothman, 2013). Here, we opted to focus on studies investigating insular and cingulate cortex in order to remain within a comparable framework in terms of investigated brain areas. In this report, considerable effort was put into a correct anatomical classification of the regions of interest and the corresponding voxel positioning. Unfortunately, in neuroimaging studies, there is still no overarching rationale that supports identical basic definition regimes with respect to a distinct classification of cortical and subcortical subunits. This fact is reflected even in actual reports as rather imprecise anatomical descriptions are used (e.g., reported is “insula activity” despite the activity is clearly located in “anterior insula subdivision”). The importance of a correct sub-classification was demonstrated by a study conducted by Gutzeit et al. (2013) showing significant differences in basic neurochemical concentrations between left and right lateralized anterior/posterior insular subdivisions. The differences were robust during acute dental pain stimulation, presumably reflecting inherent functional specificities of these insular subareas.

With respect to the cingulate areas, our approach is based on extensive anatomical and cytoarchitectonic work (see introduction and methods section for all references incorporated). We consider that Dou et al. (2013) are partly in line with the presented anatomical definition, meaning that their investigated cingulate subareas, namely pgACC and aMCC, are almost identical to ours. Yet, the difficulty in comparing the works refers to the fact that they utilized ultra-high fields at 7-Tesla and opted to calculate intra-class correlations (ICC, instead of coefficients of variation, CV). This value represents a measure of reliability (instead of reproducibility) and therefore depends on population heterogeneity which makes it rather difficult to compare between studies (Müller and Büttner, 1994; Henriques et al., 2013). It has to be clearly noted here that based on their reported ICC values, they were able to reach high reliability values as well, however, reporting other neurochemicals as glutamine and GABA. Both components are tedious to detect without applying specific imaging regimes at 3-Tesla.

Venkatraman et al. (2006) estimated ^1^H-MRS reproducibility aspects in left and right anterior cingulate and hippocampus at 4-Tesla. Their voxel sizes were 6 ml (compared to our 4.14 ml), thus substantially larger. Based on the information provided (transversal section of voxel positioning scheme), it is not clear how specifically they defined their investigated cingulate subarea. Interestingly, they revealed slightly higher intra-subject CVs compared with our results despite using larger voxels and higher field strengths [Mean CV (right/left hemisphere): tNAA = 10.0/7.2%; tCho = 12.8/9.1%; Cre = 9.5/6.6%; mI = 23.2/12.2%; Glu = 11.8/14.1%; Glx = 17.4/13.3%]. In addition to the different sequence parameters and water suppression scheme applied, differences in the CVs could be due to their used variability measure. Venkatraman et al. (2006) reported precision as a measure of reproducibility which was based on 10 repetitive measurements of the same subject (compared to our nine subjects and four repetitive measurements. The differences in the CVs of Venkatraman et al. (2006) and our report could be further rooted in their broader linewidth of the right cingulate spectra [ca. 0.068 ppm (right hemisphere) vs. our 0.0532 ppm], or that two different cingulate subareas were partly included, namely pgACC and aMCC. Due to variations in their cytoarchitectural structure, related neurochemistry might also differ what could be reflected in the differences reported by Venkatraman et al. (2006).

Jang et al. (2005) did reproducibility analyses in the cingulate (similar to our pgACC-volume) and insular cortices using a 3 T-scanner and voxel sizes of 4 ml. Contrary to the rationale of the present study, they did not separate between anterior and posterior segments of the insular cortex, thus, their results have to be compared to ours with caution. They revealed CV values for the inter-subject variability [tNAA (pgACC/Insula) = 8.7/9.4%; Cre = 7.6/11.4%; tCho = 11.8/13.1; mI = 13.8/15.9; Glu = 11.0/13.1; Glx = 7.2/11.2]. Interestingly, despite applying larger insular cortex voxels, the characteristic reproducibility values were slightly higher than those of our study. It can only be speculated whether this fact results from not accounting for the cytoarchitectonic differences between anterior/posterior insular compartments or due to a more heterogeneous study population. Considering the pgACC, their results indicate slightly lower inter-subject variability which might be rooted either in the larger voxel (4 ml to 3.46 ml) or attributed to the fact that they measured the pgACC of each of the 21 persons twice and averaged the intra-subject data thus reducing the intra-subject variance component in the inter-subject variability measures.

Altogether, bearing in mind differences in methodology and voxel definition, our work suggest comparable or slightly superior levels of variability in comparison to studies testing reproducibility of ^1^H-MRS in the cingulate and insular cortex (Jang et al., 2005; Venkatraman et al., 2006). These results are particularly remarkable regarding the applied voxel sizes which were used for neurochemical quantification in our regions of interest. Therefore, the present report suggests that pgACC, aMCC, pIL/pIR can well be targeted in clinical and scientific questions even using standard MR-equipment and ^1^H-MRS sequences at 3 Tesla. As pointed out in the introduction section, those areas play pivotal roles in a multitude of brain functions that are predominantly known from fMRI studies. In our opinion, ^1^H-MRS provides a complementary option by gaining information regarding neurochemical milieus. Knowing such baseline neurochemistry allows for a more thorough understanding in both, healthy and disease conditions, as in the latter, neurochemical alterations are frequently reported.

### Limitations

This report has limitations to consider. Beginning with our primary aim to assess spectra from anatomical/functional homogeneous brain areas, we would like to emphasize existing differences across subjects’ individual anatomy. The study sample consisted exclusively of 21–30 aged male participants. An adaptation of voxel dimensions may be necessary for the investigation of pgACC, aMCC pIL/pIR in female volunteers or populations with pathological brain conditions due to different brain sizes and pathology-rooted anatomical deviations. Thus, our results may not be generalizable to studies investigating such populations and further studies might be necessary.

In the same vein, the narrow age range could represent a further limitation. Studies have shown that metabolite concentrations are subject to change over the life span (Haga et al., 2009). Our results may therefore not be generalizable to studies using a study sample with different age ranges or/and age groups due to differences in the magnitude of inter-subject variability.

A clear limitation has to be allocated in possible movement influences especially when small areas are targeted. Although using a fixations system (Pearltec Crania) we additionally quantified head movements to estimate voxel displacement between the spectroscopic acquisitions only, hence, intra-measurement movements were not accessible with this approach. However, the small peak linewidths combined with related minute variation indicate rather small movements during acquisitions, possibly related with the choice and positioning of the voxels clearly within the anatomical borders of the targeted brain subareas.

## Conclusion

Our study provides information regarding measurement reproducibility of the neurochemical milieu within strictly determined cingulate and insular subareas, namely: pregenual anterior/anterior midcingulate as well and left and right posterior insular cortices. All areas were defined according to anatomical/cytoarchitectonic work to guarantee the best possible within region homogeneity as well as an optimal agreement according to their functional specificity. It can be concluded, that the reported neurochemicals are reliably measurable in those areas as reflected in small intra/inter subject variations. We therefore think that based on the presented rationale, it is possible to conduct ^1^H-MRS studies in small and functional homogeneous – and often critically located – brain areas. When applied with the necessary diligence, we think that profound insights of the neurochemical status in both, health and disease can be obtained, possibly even on single-subject level.

## Author Contributions

Conceived and designed the study: NdM, MB, MW, and DM. Performed the experiments: NdM and MB. Analyzed the data: NdM, MB, LM, and DM. Discussed and interpreted the data: NdM, LM, MW, DM, AG, DE, and MB. Wrote first draft of the paper: NdM and MB. Revised the manuscript and approved the final version: NdM, LM, MW, DM, AG, DE, and MB.

## Conflict of Interest Statement

The authors declare that the research was conducted in the absence of any commercial or financial relationships that could be construed as a potential conflict of interest.

## Footnotes

1 http://www.fil.ion.ucl.ac.uk/spm
